# *In vitro* and *in silico* studies of bis (indol-3-yl) methane derivatives as potential α-glucosidase and α-amylase inhibitors

**DOI:** 10.1080/14756366.2021.1971976

**Published:** 2021-08-30

**Authors:** Peng-Fei Zheng, Zhuang Xiong, Cui-ying Liao, Xin Zhang, Mei Feng, Xiao-Zheng Wu, Jing Lin, Lin-Sheng Lei, You-Cheng Zhang, Shao-Hua Wang, Xue-Tao Xu

**Affiliations:** a Second Hospital of Lanzhou University, Lanzhou, PR China; b School of Biotechnology and Health Sciences, Wuyi University, Jiangmen, PR China; c School of Pharmacy & State Key Laboratory of Applied Organic Chemistry, Lanzhou University, Lanzhou, PR China

**Keywords:** Bis (indol-3-yl) methanes, α-Glucosidase, α-Amylase, inhibitor, molecular docking

## Abstract

In this paper, bis (indol-3-yl) methanes (BIMs) were synthesised and evaluated for their inhibitory activity against α-glucosidase and α-amylase. All synthesised compounds showed potential α-glucosidase and α-amylase inhibitory activities. Compounds **5 g** (IC_50_: 7.54 ± 1.10 μM), **5e** (IC_50_: 9.00 ± 0.97 μM), and **5 h** (IC_50_: 9.57 ± 0.62 μM) presented strongest inhibitory activities against α-glucosidase, that were ∼ 30 times stronger than acarbose. Compounds **5 g** (IC_50_: 32.18 ± 1.66 µM), **5 h** (IC_50_: 31.47 ± 1.42 µM), and **5 s** (IC_50_: 30.91 ± 0.86 µM) showed strongest inhibitory activities towards α-amylase, ∼ 2.5 times stronger than acarbose. The mechanisms and docking simulation of the compounds were also studied. Compounds **5 g** and **5 h** exhibited bifunctional inhibitory activity against these two enzymes. Furthermore, compounds showed no toxicity against 3T3-L1 cells and HepG2 cells.

**Highlights**
1. A series of bis (indol-3-yl) methanes (BIMs) were synthesised and evaluated inhibitory activities against *α*-glucosidase and α-amylase.
2. Compound **5g** exhibited promising activity (IC_50_ = 7.54 ± 1.10 μM) against *α*-glucosidase.
3. Compound **5s** exhibited promising activity (IC_50_ = 30.91 ± 0.86 μM) against α-amylase.
4. In silico studies were performed to confirm the binding interactions of synthetic compounds with the enzyme active site.

## Graphical Abstract

**Figure:**
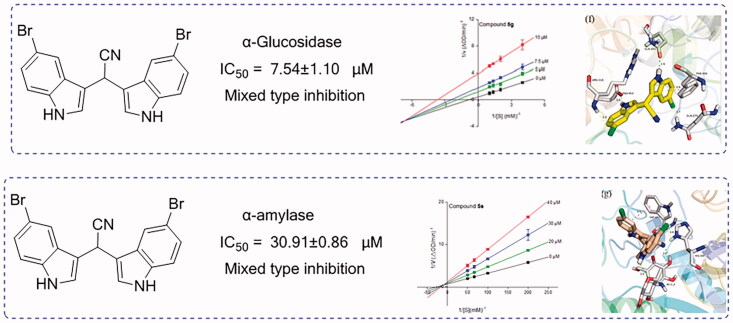

## 1. Introduction

Diabetes mellitus (DM) is one of common metabolic disease characterised by hyperglycaemia.^1^ The main clinical treatment strategies for DM is to control the blood glucose level using drugs.^2^ The catalytic hydrolysis of carbohydrates by enzymes such as α-glucosidase and α-amylase is the most important reason for the increase of glucose in blood.^3^

α-Glucosidase(EC 3.2.1.20), existing in the surface of small intestine, is an important hydrolase enzyme.^4–5^ It catalyses the hydrolysis of carbohydrates into absorbable glucose monomers by spliting the bond between glucosidic oxygen and glucosyl residues of carbohydrates.^6–7^ α-Amylase (E.C.3.2.1.1), one hydrolase enzyme, is mainly secreted by the pancreas and salivary glands.^8–9^ α-Amylase catalytic hydrolyse the starch to produces maltose and glucose by breaking α-1,4-glucosidic bonds.^10–11^ The inhibition the activity of α-glucosidase or α-amylase delay the hydrolase of polysaccharides, consequently, the postprandial blood glucose level can be reduced, which is believed as an effective approach for the treatment strategy of DM. To date, a large amount of α-glucosidase and α-amylase inhibitors are obtained from natural products and chemical synthesis.^12–15^ However only few have further application, such as acarbose, voglibose, and miglitol. However, these clinical drugs are often associated with side-effects. Therefore, it is still worth further investigation for the development of more effective inhibitors towards α-glucosidase and α-amylase.

Bis (indol-3-yl) methanes (BIMs), as the key skeletons, present in a variety of bioactive natural products isolated from marine organisms, land plants and microorganisms.^16–18^ And such a fact has also stimulated the synthesis of different BIMs leading to the reveal of a wide range of bio-pharmacological activities, including anti-fungal, anti-inflammatory, anti-oxidant, anti-cancer, and antibacterial activities.^19–22^ Especially, it is notable that BIMs are reported to process activities of lowering blood lipids and preventing obesity^23^ as well as inhibiting α-glucosidase activity, showing great potential in the treatment of DM. In order to find potential α-glucosidase inhibitors, some BIMs were synthesised and presented effective inhibitory activity.^24–25^

The cyano group, a carbon-nitrogen triple bond, has been widely used in the structural modification and transformation of small drug molecules. It can change the physical and chemical properties of small molecules, enhance the interaction between drug molecules and target proteins to improve drug efficacy, improve the metabolic stability of compounds in the body, and so on.^26^ The cyano group also is used as an important substitution group in some reported α-glucosidase inhibitors.^27–28^

Based on the principle of combination of active structural moieties in drug design and our synthetic methodology for BIMs derivatives,^29^ we synthesised BIMs derivatives to evaluate them for their α-glucosidase and α-amylase inhibitory activity.

## 2. Results and discussion

### 2.1. Chemistry

**Scheme 1:**
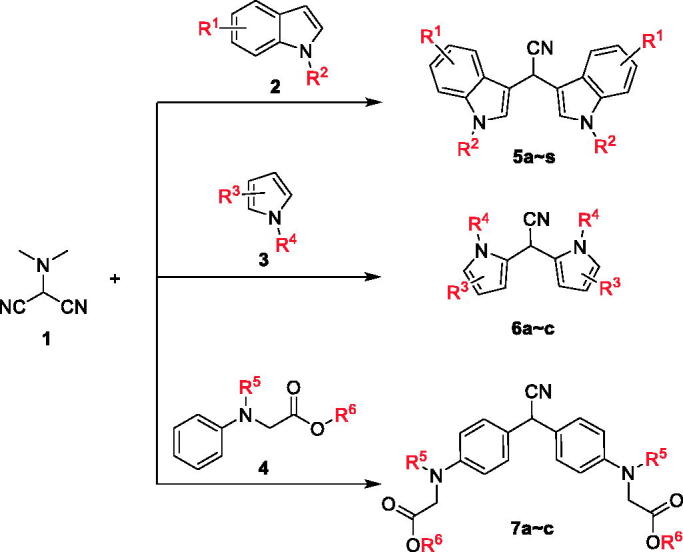
Synthesis of BIMs. Reagents and conditions: Al(OTf)_3_ (0.2 equiv.), DCE, 120 °C, 8 h.

Based on the effective synthetic method of BIMs reported by our group, **5a ∼ c**, **6a ∼ c**, and **7a ∼ c** were synthesised from *N,N*-dimethylaminomalononitrile (**1**) with substituted indole (**2**), substituted pyrrole (**3**) or *N*-phenylaminoacetic acid ethyl ether (**4**) under the catalysis of Al(OTf)_3_. The synthetic route was shown in Scheme 1. All synthetic compounds are known compounds, which were reported in references.^29^

### 2.2. Evaluation of activity against α-glucosidase

#### 2.2.1. α-Glucosidase inhibition assay

The *in vitro* α-glucosidase inhibitory activities of all synthesised compounds were evaluated using *p*-nitrophenyl-α-D-glucopyranoside (PNPG) as substrate. As shown in Table 1, BIMs **5a ∼ c** (IC_50_: 7.54 ± 1.10 to 150.48 ± 3.16 μM) presented good inhibitory activities, stronger than acarbose (IC_50_: 261.45 ± 2.17 μM). Among them, **5 g**, **5e**, and **5 h** displayed strongest inhibitory activities with the IC_50_ values of 7.54 ± 1.10, 9.00 ± 0.97 and 9.57 ± 0.62 µM, respectively, those were ∼ 30 times stronger than that of acarbose. While, **6a ∼ c**, **7a ∼ c** showed moderate inhibitory activities with IC_50_ values from 100.38 ± 0.53 to 242.78 ± 5.14 µM.

**Table 1.** α-Glucosidase and α-amylase inhibitory activity of all synthesised compounds.

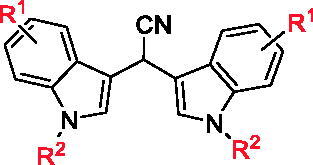				
Compounds	R^1^	R^2^	IC_50_ (µM) on α-glucosidase	IC_50_ (µM) on α-amylase
**5a**	H	H	55.53 ± 0.40	493.59 ± 10.34
**5b**	5-F	H	40.86 ± 0.27	147.09 ± 6.23
**5c**	4-Cl	H	45.10 ± 1.42	151.25 ± 5.21
**5d**	5-Cl	H	10.22 ± 0.63	41.95 ± 1.97
**5e**	6-Cl	H	9.00 ± 0.97	40.03 ± 2.14
**5f**	7-Cl	H	20.65 ± 0.42	266.86 ± 3.87
**5g**	5-Br	H	7.54 ± 1.10	32.18 ± 1.66
**5h**	2-Ph	H	9.57 ± 0.62	31.47 ± 1.42
**5i**	2-CH_3_	H	120.83 ± 4.45	123.44 ± 3.27
**5j**	4-CH_3_	H	70.53 ± 1.42	151.60 ± 2.94
**5k**	5-CH_3_	H	55.99 ± 0.78	406.31 ± 8.44
**5l**	6-CH_3_	H	50.64 ± 1.78	36.35 ± 1.37
**5m**	7-CH_3_	H	150.48 ± 3.16	500.41 ± 13.57
**5n**	5-F,2-CH_3_	H	50.97 ± 0.69	154.05 ± 3.08
**5o**	5-CH_3_,2-CH_3_	H	65.06 ± 0.61	472.77 ± 7.65
**5p**	5-OCH_3_	H	50.37 ± 1.06	137.81 ± 5.62
**5q**	5-COOCH_3_	H	80.12 ± 0.79	205.36 ± 4.87
**5r**	H	CH_3_	100.96 ± 1.69	153.04 ± 3.43
**5s**	5-Br	CH_3_	30.48 ± 1.27	30.91 ± 0.86
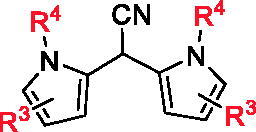				
Compounds	R^3^	R^4^	IC_50_ (µM)	
**6a**	H	CH_3_	220.31 ± 5.02	463.96 ± 9.47
**6b**	2-CH_3_	H	120.77 ± 3.51	435.62 ± 10.65
**6c**	H	H	130.36 ± 3.81	418.71 ± 11.32
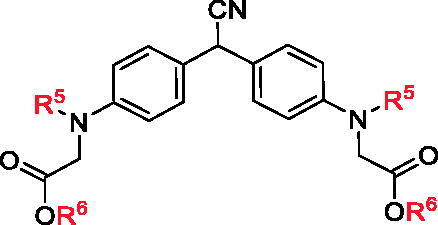				
Compounds	R^5^	R^6^	IC_50_ (µM)	
**7a**	CH_3_	CH_2_CH_3_	100.38 ± 0.53	510.16 ± 12.55
**7b**	H	CH_2_CH_3_	180.05 ± 0.79	139.21 ± 4.85
**7c**	H	CH_3_	242.78 ± 5.14	479.17 ± 13.44
**Acarbose**			261.45 ± 2.17	80.33 ± 2.95

#### 2.2.2. Structure activity relationships (SAR) analysis

**Figure 1.**
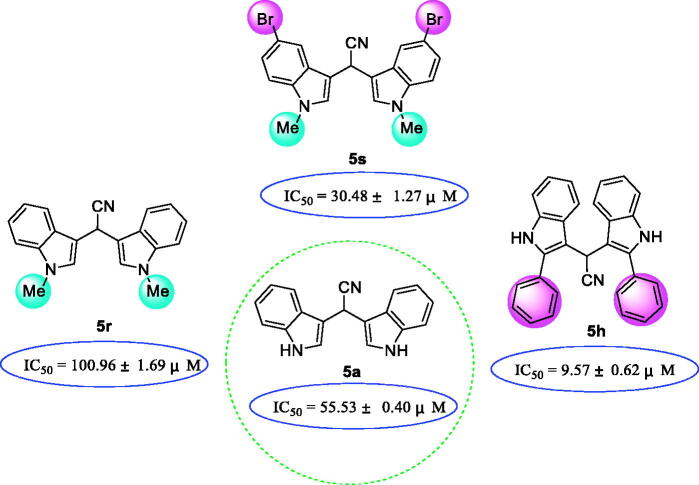
SAR analysis of compounds **5a**, **5r**, and **5 s**.

The SAR of compounds was discussed by analysing the substitution pattern on indole moiety. Compound **5a** (IC_50_ = 55.53 ± 0.40 µM) with no substitution on indole ring showed ∼5 fold stronger compared to acarbose (IC_50_ = 261.45 ± 2.17 µM). Compound **5r** (IC_50_ = 100.96 ± 1.69 µM) with a methyl group at 1-position of indole presented decreased activity. However, introducing bromine group on the indole ring of **5r**, compound **5 s** (IC_50_ = 30.48 ± 1.27 µM), caused obviously increase in inhibition activity. The introduction of two phenyl groups at the 2-position of indole ring (compound **5 h**, IC_50_ = 9.57 ± 0.62 µM) resulted in markedly increase of activity (Figure 1). The above results indicated possible hydrogen bond interaction between compound **5a** and α-glucosidase, halogen bond between **5 s** and glucosidase, as well strong π-π stacking effect between **5 h** and α-glucosidase.

**Figure 2.**
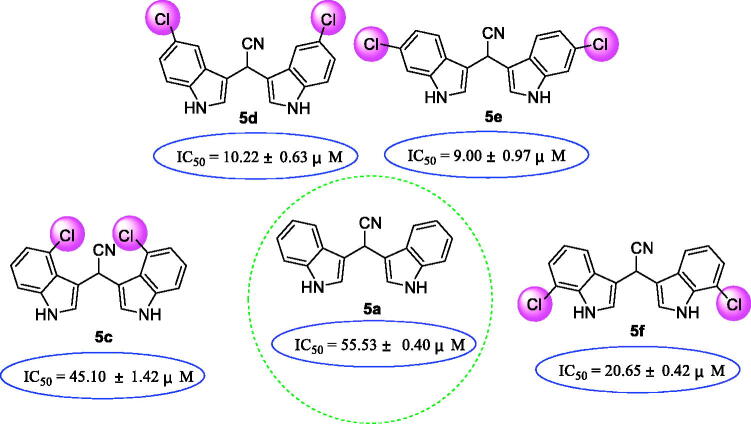
SAR analysis of compounds **5a**, **5c∼5f**.

Compared to compound **5a**, compounds **5c∼5f** with chlorine substituent on the benzene ring of indole had more potent activities. This indicated that chlorine atom, a typical halogen atom, might be well interact with α-glucosidase through halogen bond like that of compound **5 s** (Figure 2). Among chlorine substituted derivatives, **5e** (IC_50_ = 9.00 ± 0.97 µM) with the 6-chlorine group was found to be most active and 29 times better activity than acarbose. It’s positional isomer **5d** (IC_50_ = 10.22 ± 0.63 µM) with 5-chlorine group, presented similar activity. However, **5c** (IC_50_ = 45.10 ± 1.42 µM) and **5f** (IC_50_ = 20.65 ± 0.42 µM) with 4-chlorine group and 7-chlorine group respectively showed lower activities.

**Figure 3.**
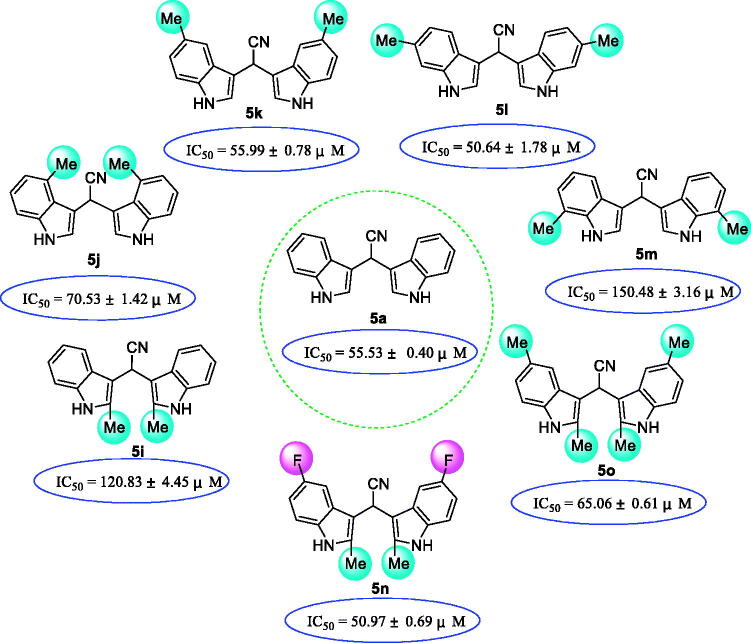
SAR analysis of compounds **5a**, and **5i ∼ o**.

Compounds **5i ∼ o** with methyl group on the indole ring showed moderate inhibitory activities. This indicated that methyl group, an electron donating group, had no beneficial effects on the interaction with α-glucosidase (Figure 3). Compared to compound **5a**, introduction of 6-methyl group **5 l** (IC_50_ = 50.64 ± 1.78 µM) and 5-methyl group **5k** (IC_50_ = 50.64 ± 1.78 µM) resulted in slight changes on inhibitory activities, while presence of 2-methyl group **5i** (IC_50_ = 120.83 ± 4.45 µM), 4-methyl group **5i** (IC_50_ = 70.53 ± 1.42 µM), 7-methyl group **5 m** (IC_50_ = 150.48 ± 3.16 µM), and 2,5-dimethyl groups **5o** (IC_50_ = 65.06 ± 0.61 µM) caused obvious decrease on the activity. When introducing 5-fluorine group, compound **5n** (IC_50_ = 50.97 ± 0.69 µM) and 5-methyl group, compound **5o** (IC_50_ = 65.06 ± 0.61 µM) showed visible increase on the inhibitory activity, as compared to compound **5i**. This might be attributed to possible hydrogen bond interaction formed by fluorine atom.

**Figure 4.**
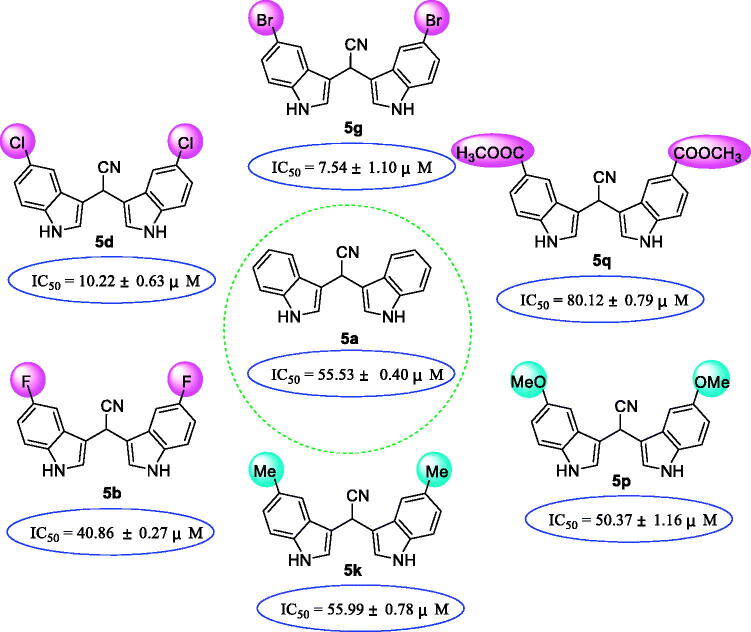
SAR analysis of compounds **5a**, **5 b**, **5d**, **5 g**, **5q**, **5p**, and **5k**.

Analysing effect of different type of substituent at same substituent position (Figure 4), halogen atom substituents lead to strengthen of α-glucosidase inhibitory activities, while electron donating and withdrawing groups resulted in decrease of the activity, as compared to compound **5a**. Compound **5 g** (IC_50_ = 7.54 ± 1.10 µM) with 5-bromine groups presented 34 times stronger activity than acarbose, and **5 b** (IC_50_ = 40.86 ± 0.27 µM) with 5-fluorine groups displayed 6 times stronger activity than acarbose. However, compound **5k** (IC_50_ = 55.99 ± 0.78 µM) with 5-methyl group and **5p** (IC_50_ = 50.37 ± 1.06 µM) with 5-methoxy group exhibited lower activities than that of **5a**. In addition, the introduction of electron withdrawing group, methyl formate, **5q** (IC_50_ =80.12 ± 0.79 µM) resulted in a decrease on the α-glucosidase inhibitory activity.

Compared to series of **5a**∼**s**, **6a**∼**c** and **7a**∼**c** only presented moderate α-glucosidase inhibitory activities with IC_50_ values from 100.38 ± 0.53 µM to 242.78 ± 5.14 µM, indicating that indole ring might be more beneficial than pyrrole ring and benzene ring on α-glucosidase inhibitory activities.

#### 2.2.3. Inhibition mechanism study of α-glucosidase inhibitor

**Figure 5.**
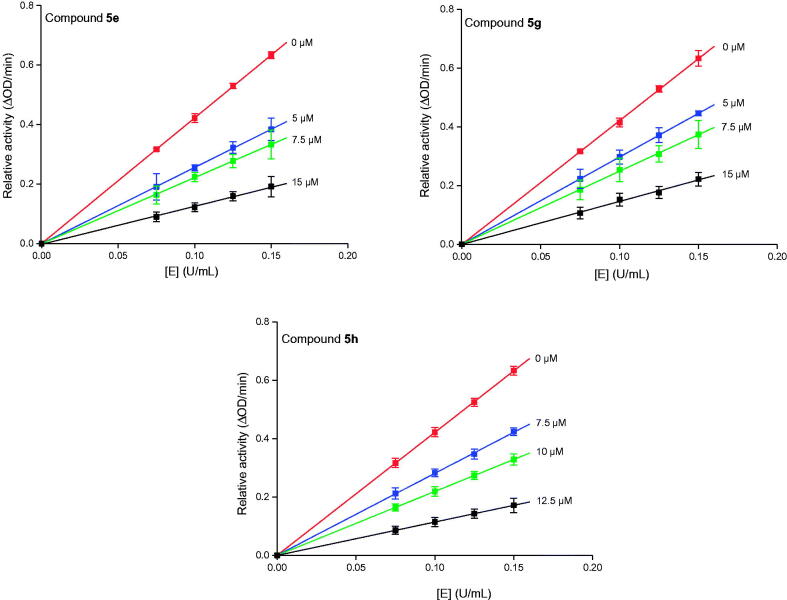
The plots of α-glucosidase concentration *vs* enzyme activity in presence of compounds **5e**, **5 g**, and **5 h**.

To gain further insight into the interaction between BIMs and α-glucosidase, the inhibition mechanism of compounds **5e**, **5 g**, and **5 h** with highly potent inhibitory were investigated. The relationship of enzyme concentration with enzyme activity in presence of compounds **5e**, **5 g**, and **5 h** was firstly investigated. As shown in Figure 5, the plots of enzyme concentration *vs* remaining enzyme activity at different inhibitor concentrations give a group of straight lines that all passed through the origin point. Those results indicated that the α-glucosidase inhibition of compounds **5e**, **5 g**, and **5 h** was reversible.

**Figure 6.**
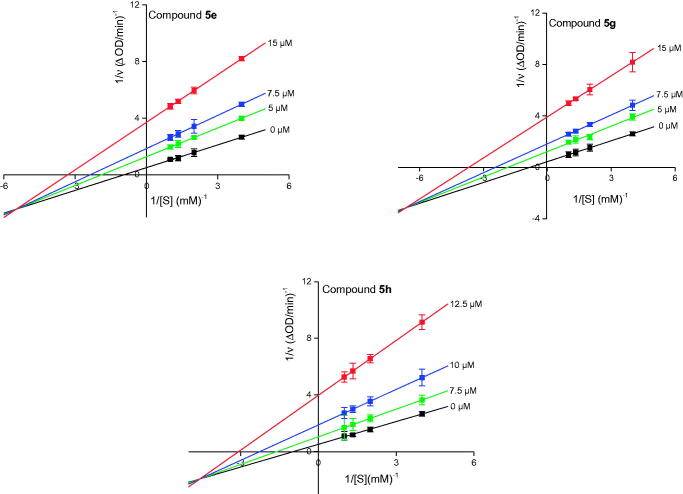
Lineweaver-Burk plots of α-glucosidase inhibition by compounds **5e**, **5 g**, and **5 h**.

The inhibitory kinetics of compounds **5e**, **5 g**, and **5 h** on α-glucosidase were studied using Lineweaver-Burk plots. For compounds **5e**, **5 g**, and **5 h**, the plots of 1/*v vs* 1/[*S*] give a lot of straight lines that intersected at the same point in the third quadrant respectively (Figure 6), revealing that compounds **5e**, **5 g**, and **5 h** were all mixed-type inhibitors. These results indicated that compounds **5e**, **5 g**, and **5 h** could bind with free enzyme, as well as, enzyme-substrate complex to reduce the catalytic activity of α-glucosidase.

Besides, we also determined the equilibrium constants of binding of inhibitors to free enzymes (*K*_I_) and enzyme-substrate complexes (*K*_IS_) through plots of slope (*K*_m_*/V*_m_) and vertical intercept (1*/V*_m_) *vs* inhibitor concentration. The results were presented in Table 2. The *K*_I_ values of compounds **5e**, **5 g**, and **5 h** were higher than their *K*_IS_ values, suggesting that the affinity of compounds **5e**, **5 g**, and **5 h** with free enzyme was lower than that with enzyme-substrate complex.

**Table 2.** The inhibition type, as well as *K*_I_ and *K*_IS_ values of **5e**, **5 g**, and **5 h** against α-glucosidase.

Compound	Inhibition type	*K*_I_ value (μM)	*K*_IS_ value (μM)
**5e**	Mixed type	13.36	2.11
**5g**	Mixed type	14.93	1.59
**5h**	Mixed type	8.71	1.69

#### 2.2.4. Molecular docking study

**Figure 7.**
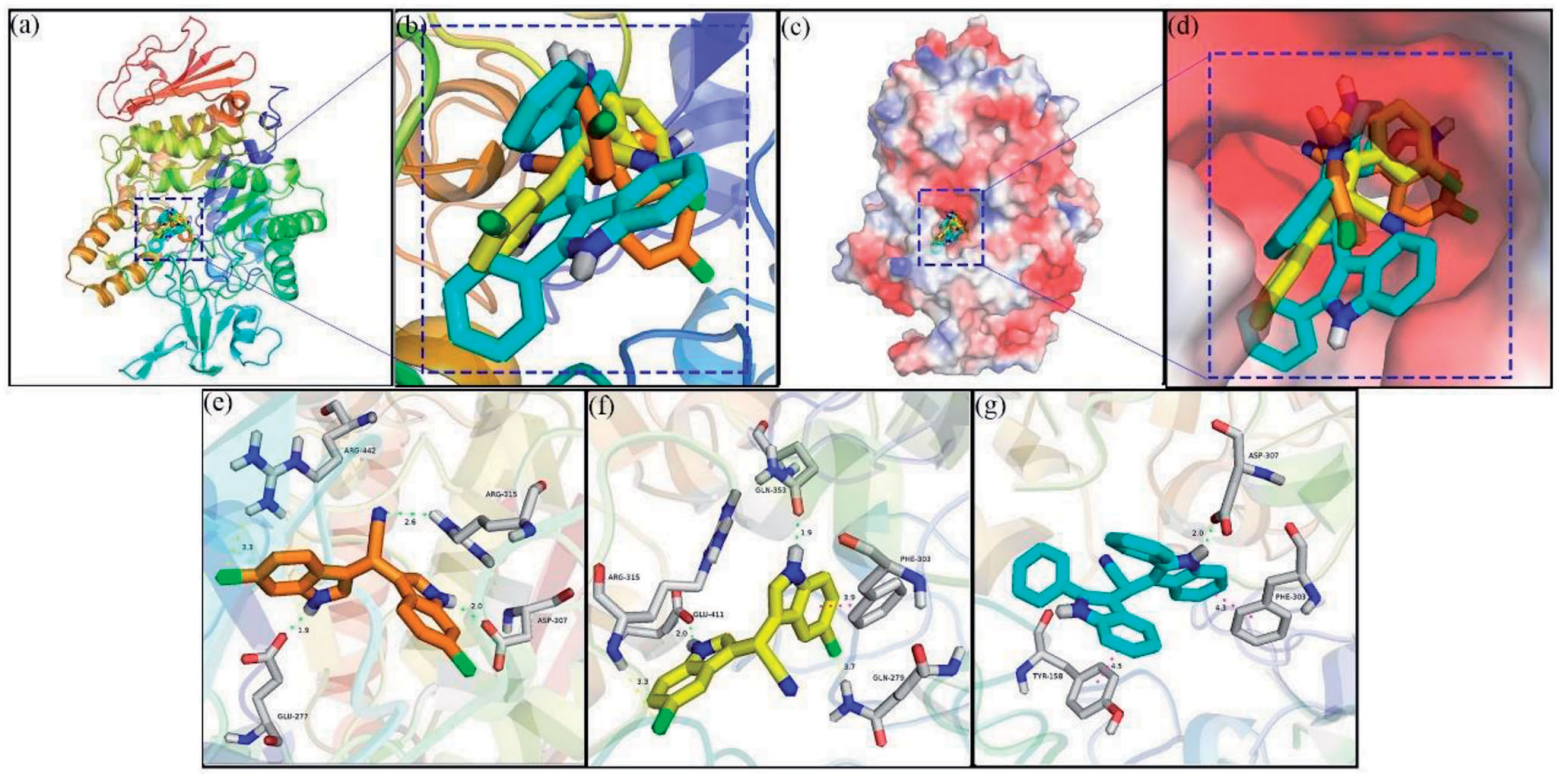
Molecular docking of compounds **5e**, **5 g**, and **5 h** with α-glucosidase.

Sybyl molecular docking program was used to simulate the binding modes of α-glucosidase with the topmost active compounds, i.e. **5e**, **5 g**, and **5 h** to further understand the inhibition mechanism. As shown in Figure 7(a–d), compound **5e** (orange compound), **5 h** (yellow compound), and **5 g** (cyans compound), were well nested into the active site of α-glucosidase and presented similar coordination with the active site of enzyme. The docking of compound **5e** was presented in Figure 7(e). One indole ring nitrogen formed a hydrogen bond (2.0 Å) with Asp307, another indole ring nitrogen formed a hydrogen bond (1.9 Å) with Glu277, and cyano nitrogen formed a hydrogen bond (2.6 Å) with Arg315. Also, chlorine on indole ring formed a halogen bond with Arg442 (3.3 Å). For compound **5 g** (Figure 7(f)), The two indole ring nitrogen made two hydrogen bonds with Gln353 (1.9 Å) and Glu411 (2.0 Å), respectively, and the two chlorines on indole ring made two halogen bond with Arg315 (2.0 Å) and Gln279 (3.7 Å), respectively. Moreover, one indole ring made π-π interactions with Phe303 (3.9 Å). Compounds **5 h** (Figure 7(g)) also formed a hydrogen bond with Asp307 (2.0 Å), and formed π-π interactions with Phe303 (4.3 Å) and Tyr158 (4.5 Å), respectively. The hydrogen bond, π-π interactions, or halogen bond existed between α-glucosidase with compounds **5e**, **5 g**, or **5 h** played very important roles in the binding of compounds and proteins.

### 2.3. *Evaluation of activity against* α-amylase

#### 2.3.1. α-Amylase inhibition assay

Meantime, all compounds were tested for α-amylase inhibitory activity *in vitro* and their IC_50_ values were recorded in Table 1. All synthesised BIMs displayed good to moderate inhibitory activities, IC_50_ range of 30.91 ± 0.86 ∼ 510.16 ± 12.55 µM. Excitingly, compounds **5 g** (IC_50_: 32.18 ± 1.66 µM), **5 h** (IC_50_: 31.47 ± 1.42 µM), and **5 s** (IC_50_: 30.91 ± 0.86 µM) showed strongest inhibitory activities, ∼ 2.5 times stronger than that of acarbose (IC_50_: 80.33 ± 2.95 µM).

#### 2.3.2. Sar analysis

The SAR of all compounds against α-amylase were also analysed with compound **5a** (IC_50_: 493.59 ± 10.34 µM) as template compound. The introduction of halogen (F, Cl, Br) on indole ring effectively increased the inhibitory activity, Br group (compound **5 g**, IC_50_: 32.18 ± 1.66 µM) is most beneficial for the inhibitory activity, and Cl group at 6-position (compound **5e**, IC_50_: 40.03 ± 2.14 µM) of indole presented better inhibitory activity compared to other position. The introduction of methyl group also changed the inhibitory activity, and compound **5 l** with methyl group at 6-position (IC_50_: 36.35 ± 1.37 µM) presented the best inhibitory activity. The introduction of methoxy, Phenyl, and ester group also increased the inhibitory activity, especially, compound **5 h** with Phenyl group (IC_50_: 31.47 ± 1.42 µM) extended good inhibitory activity. Furthermore, compounds **6a**∼**c** and **7a**∼**c** presented effectively lower α-amylase inhibitory activities than compounds **5a**∼**s**, indicating that indole ring was more helpful for α-amylase inhibitory activities.

#### 2.3.3. Inhibition mechanism study of α-amylase inhibitor

**Figure 8.**
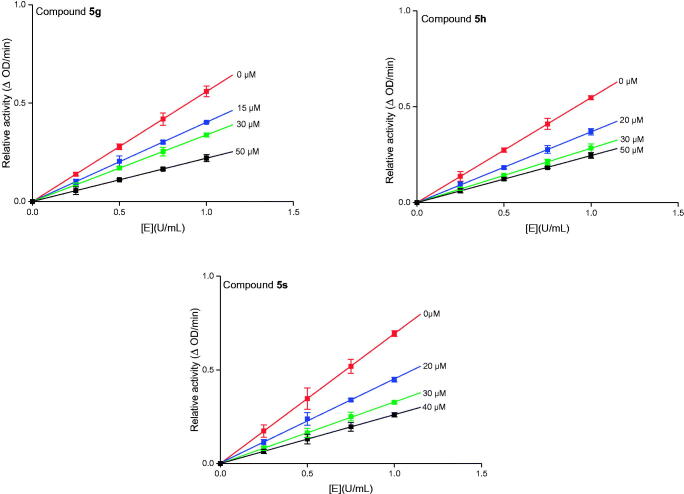
The relationship of α-amylase concentration with α-amylase activity in presence of compounds **5 g**, **5 h**, and **5 s**.

**Figure 9.**
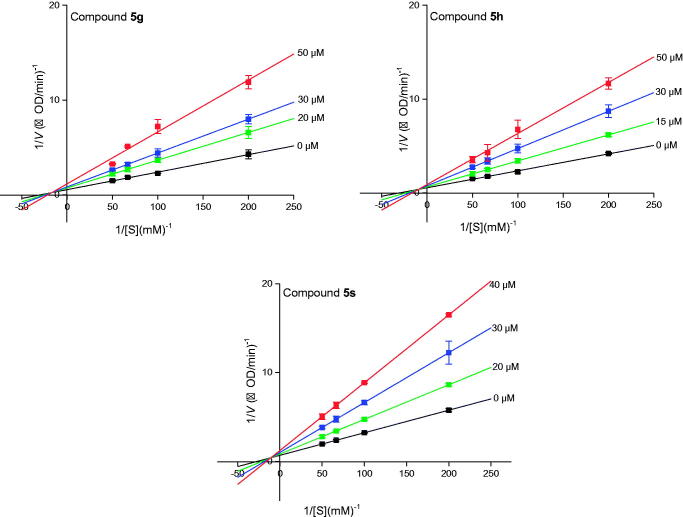
Lineweaver-Burk plots of α-amylase inhibition by compounds **5e**, **5 g**, and **5 h**.

The highest potent inhibitory compounds **5 g**, **5 h**, and **5 s** were evaluated their inhibition mechanism. As shown in Figure 8, the remaining enzyme activity at different inhibitor concentrations was measured and the obtained straight lines passed through the origin point, suggesting that compounds **5 g**, **5 h**, and **5 s** reversibly inhibited the α-amylase. Lineweaver-Burk plots were used to analyse the inhibitory kinetics of compounds **5 g**, **5 h**, and **5 s**. The plots of 1/*v vs* 1/[*S*] intersected a point at the second quadrant, indicating that **5 g**, **5 h**, and **5 s** were mixed inhibitors (Figure 9). Besides, the *K*_I_ and *K*_IS_ values of compounds **5 g**, **5 h**, and **5 s** were measured, showing *K*_I_ values were higher than *K*_IS_ values (Table 3).

**Table 3.** The inhibition type, as well as *K*_I_ and *K*_IS_ values of **5 g**, **5 h**, and **5 s** against α-amylase.

Compound	Inhibition type	*K*_I_ value (μM)	*K*_IS_ value (μM)
**5g**	Mixed type	78.33	25.02
**5h**	Mixed type	37.39	1.63
**5s**	Mixed type	25.98	9.01

#### 2.3.4. Docking simulation for α-amylase

**Figure 10.**
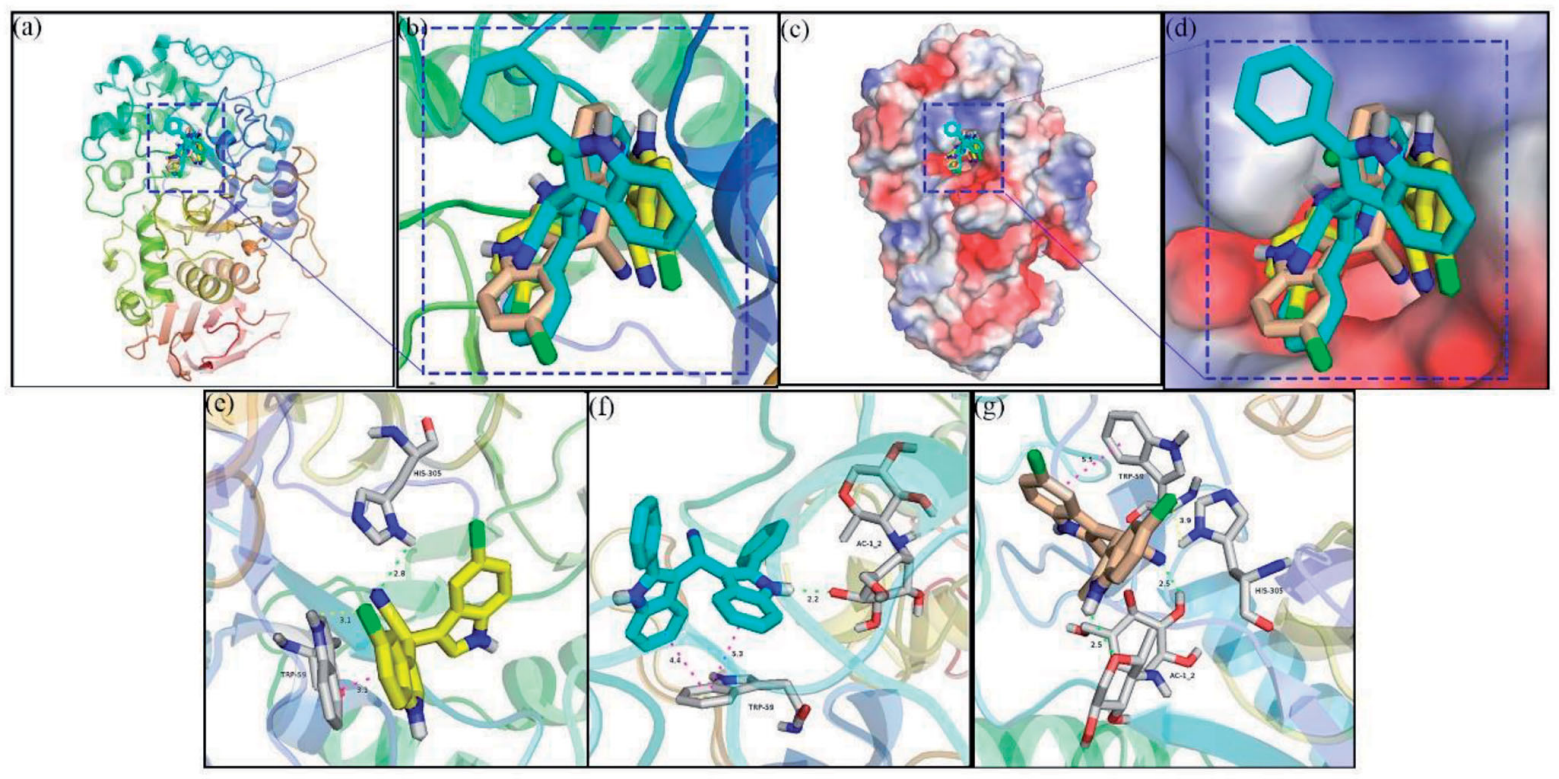
Molecular docking of compounds **5 g**, **5 h**, and **5 s** with α-amylase.

As mentioned above, molecular docking between compounds **5 g**, **5 h**, and **5 s** with α-amylase were used to explain their mechanism. Docking results revealed that compounds **5 g** (yellow compound), **5 h** (cyang compound), and **5 s** (wheat compound) were well located within the active site of α-amylase (Figure 10(a–d)). Binding models of compounds **5 g**, **5 h**, and **5 s** with α-amylase were depicted in Figure 10(e–f), respectively. For compound **5 g**, cyano nitrogen made a hydrogen bond with His305 (2.8 Å), and chlorine on indole ring made a halogen bond with Trp59 (3.1 Å) and one indole ring made π-π interactions with Trp59 (3.1 Å). The docking of compound **5e** showed that one indole ring nitrogen formed a hydrogen bond (2.2 Å) with Ac1_2, and two indole rings formed π-π interactions with Trp59 (4.4 and 5.3 Å). The indole ring nitrogen and cyano nitrogen of compounds **5 s** also formed two hydrogen bonds with Ac1_2 (2.5 and 2.5 Å), a hydrogen bond with His305 (3.9 Å), and π-π interactions with Trp59 (5.5 Å), respectively. The hydrogen bond, π-π interactions, or halogen bond existed between α-amylase with compounds **5 g**, **5 h**, or **5 s** were helpful to stabilise the formation of the ligand-protein complex.

Based on above assays results of compounds targeting α-glucosidase and α-amylase, compounds **5e**, **5 g**, and **5 h** showed highest α-glucosidase inhibitory and compounds **5 g**, **5 h**, and **5 s** presented highest α-amylase inhibitory. Thence, compounds **5 g** and **5 h** displaying the potential bifunctional could be considered as the lead compounds for bifunctional drugs.

### 2.4. In vitro cytotoxicity assay

**Figure 11.**
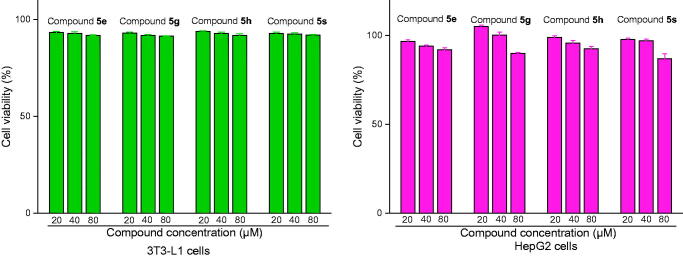
Cytotoxicity assay of compounds **5e**, **5 g**, **5 h**, and **5 s** on 3T3-L1 cells and HepG2 cells.

Moreover, the preliminary *in vitro* safety of compounds **5e**, **5 g**, **5 h**, and **5 s** were tested on Mouse Preadipocytes cells (3T3-L1) and Human liver cancer cells (HepG2) using MTT method and the results were shown in Figure 11. Compounds **5e**, **5 g**, **5 h**, and **5 s** displayed no effect on the viability of 3T3-L1 cells and HepG2 cells at concentration range of 2 0 ∼ 80 μM, suggesting these promising inhibitors had non-toxic towards live cells.

## 3. Conclusion

For searching α-glucosidase and α-amylase inhibitor, a variety of synthetic BIMs were prepared and assayed. Compounds **5 g** (IC_50_: 7.54 ± 1.10 μM), **5e** (IC_50_: 9.00 ± 0.97 μM), and **5 h** (IC_50_: 9.57 ± 0.62 μM) were the strongest inhibitor against α-glucosidase and compounds **5 g** (IC_50_: 32.18 ± 1.66 µM), **5 h** (IC_50_: 31.47 ± 1.42 µM), and **5 s** (IC_50_: 30.91 ± 0.86 µM) were the strongest inhibitor against α-amylase. The mechanisms results revealed that compounds were reversible mixed-type inhibitors. Molecular docking explained the action mechanism between inhibitors and target proteins. Finally, their low toxicity to 3T3-L1 cells and HepG2 cells lays the foundation for *in vivo* studies.

## 4. Materials and methods

### 4.1. Chemistry

α-Glucosidase, α-amylase, 4-Nitrophenyl-β-D-galactopyranoside (PNPG) and 3–(4,5-Dimethythiazol-2-yl)-2,5-diphenyl-tetrazoliumbromide (MTT) were supplied by Sigma-Aldrich. Water-soluble starch was obtained from Shanghai Yuanye Biological Technology Co.,Ltd. 3T3-L1 cells and HepG2 cells were supplied by ATCC. Dulbecco’s Modified Eagle’s Medium (DMEM), Foetal bovine serum (FBS), penicillin and streptomycin were obtained from Gibco. All commercially available compounds were used without further purification. All reactions under standard conditions were carried out under argon and dry atmosphere.^1^H and ^13 ^C spectra were recorded in CDCl_3_, DMSO-*d*_6_, Acetone-*d*_6_ on 400 MHz instruments and spectral data were reported in ppm. High-resolution mass spectral analysis (HRMS) data were measured on the Apex II by means of the ESI technique.

### 4.2. *ENERAL procedure for the preparation of derivatives* (5a ∼ c, 6a ∼ c,7a ∼ c)

*N,N*-dimethylaminomalononitrile (**1**) (1.2 mmol), substituted indole (**2**) (1 mmol), and Al(OTf)_3_ (0.20 mmol) were added into DCE (2 ml), then the mixture was stirred at 120 °C under argon atmosphere until the reaction was complete (monitored by TLC). The mixture was evaporated to give the crude product, followed purification to produce **5a**∼**c**. Likewise, compounds **6a**∼**c** were obtained from *N,N*-dimethylaminomalononitrile (**1**) and substituted pyrrole (**3**), compounds **7a**∼**c** were synthesised from *N,N*-dimethylaminomalononitrile (**1**) and *N*-phenylaminoacetic acid ethyl ether.

#### 4.2.1. 2,2-Di(1H-indol-3-yl)acetonitrile (5a)

Rf = 0.35 (petroleum ether/ethyl acetate = 2: 1); White solid; Yield 89%; m.p.: 173.9–178.3 °C; ^1^H NMR (600 MHz, DMSO-*d*_6_) δ 11.17 (s, 2H), 7.59 (d, *J* = 7.8 Hz, 2H), 7.43–7.41 (m, 4H), 7.12 (t, *J* = 7.8 Hz, 2H), 7.00 (t, *J* = 7.8 Hz, 2H), 6.08 (s, 1H); ^13 ^C NMR (150 MHz, DMSO-*d*_6_) δ 136.54, 125.20, 123.69, 121.52, 120.68, 118.87, 118.49, 111.84, 109.43, 25.29; HRMS (ESI) calcd for C_18_H_13_N_3_ [M + Na]^+^: 294.1002, found 294.1013; IR ν (cm^−1^): 3411, 3057, 2924, 2854, 2242, 1620, 1457, 1338, 1097, 743.

#### 4.2.2. 2,2-Bis(5-fluoro-1H-indol-3-yl)acetonitrile (5 b)

Rf = 0.41 (petroleum ether/ethyl acetate = 1: 1); Yellow solid; Yield 89%; m.p.: 182.7–187.5 °C; ^1^H NMR (400 MHz, Acetone-*d*_6_) δ 10.51 (s, 2H), 7.57 (d, *J* = 2.0 Hz, 2H), 7.46 (q, *J* = 4.4 Hz, 2H), 7.28 (dd, *J* = 2.0, 9.6 Hz, 2H), 6.96–9.91 (m, 2H), 5.96 (s, 1H); ^13 ^C NMR (100 MHz, DMSO-*d*_6_) δ 156.76 (d, *J* = 230.9 Hz), 133.28, 125.89, 125.36 (d, *J* = 10.1 Hz), 120.42, 113.10 (d, *J* = 9.7 Hz), 109.91 (d, *J* = 25.9 Hz), 103.18 (d, *J* = 23.7 Hz), 25.12; HRMS (ESI) calcd for C_18_H_11_F_2_N_3_ [M + Na]^+^: 330.0813 found 330.0826; IR ν (cm^−1^): 3431, 3127, 2924, 2242, 1630, 1583, 1487, 1457, 1346, 1170, 938, 800.

#### 4.2.3. 2,2-Bis(4-chloro-1H-indol-3-yl)acetonitrile (5c)

Rf = 0.49 (petroleum ether/ethyl acetate = 1: 1); White solid; Yield 79%; m.p.:230.0–235.8 °C; ^1^H NMR (400 MHz, Acetone-*d*_6_) δ 10.68 (s, 2H), 7.46 (d, *J* = 8.0 Hz, 2H), 7.29 (d, *J* = 2.8 Hz, 2H), 7.14 (t, *J* = 7.6 Hz, 2H), 7.08 (d, *J* = 7.2 Hz), 6.87 (s, 1H); ^13 ^C NMR (100 MHz, Acetone-*d*_6_) δ 139.57, 126.95, 125.95, 123.74, 123.11, 121.71, 121.21, 112.52, 111.95, 27.78; HRMS (ESI) calcd for C_18_H_11_Cl_2_N_3_ [M + Na]^+^: 362.0222, found 362.0235; IR ν (cm^−1^): 3427, 2925, 2854, 2242, 2192, 1617, 1587, 1486, 1429, 1340, 1186, 938, 777, 764, 737.

#### 4.2.4. 2,2-Bis(5-chloro-1H-indol-3-yl)acetonitrile (5d)

Rf = 0.41 (petroleum ether/ethyl acetate =1: 1); Yellow solid; Yield 80%; m.p.: 168.0–173.4 °C; ^1^H NMR (400 MHz, CDCl_3_) δ 8.31 (s, 2H), 7.50 (d, *J* = 2.0 Hz, 2H), 7.24 (d, *J* = 8.4 Hz, 2H), 7.14– 7.10 (m, 4H), 5.48 (s, 1H); ^13 ^C NMR (100 MHz, CDCl_3_) δ 134.88, 126.18, 125.76, 124.62, 123.10, 119.52, 118.02, 112.77, 109.13, 26.05; HRMS (ESI) calcd for C_18_H_11_Cl_2_N_3_ [M + Na]^+^: 362.0222, found 362.0238; IR ν (cm^−1^): 3432, 3126, 2244, 1682, 1620, 1572, 1463, 1421, 1340, 1101, 894, 798, 737, 587.

#### 4.2.5. 2,2-Bis(6-chloro-1H-indol-3-yl)acetonitrile (5e)

Rf = 0.59 (petroleum ether/ethyl acetate =1: 1); White solid; Yield 82%; m.p.:175.6–178.2 °C; ^1^H NMR (400 MHz, DMSO-*d*_6_) δ 11.33 (s, 2H), 7.52 (d, *J* = 8.8 Hz, 2H), 7.46 (d, *J* = 2.0 Hz, 4H), 7.02 (dd, *J* = 2.0, 8.4 Hz, 2H), 6.11 (s, 1H); ^13 ^C NMR (100 MHz, DMSO-*d*_6_) δ 136.97, 126.47, 125.01, 123.94, 120.38, 119.83, 119.40, 111.57, 109.55, 25.05; HRMS (ESI) calcd for C_18_H_11_Cl_2_N_3_ [M + Na]^+^: 362.0222, found 362.0236; IR ν (cm^−1^): 3431, 3127, 2925, 2243, 1621, 1546, 1454, 1402, 1336, 1101, 1062, 907, 804, 737, 592.

#### 4.2.6. 2,2-Bis(7-chloro-1H-indol-3-yl)acetonitrile (5f)

Rf = 0.71 (petroleum ether/ethyl acetate = 1: 1); White solid; Yield 84%; m.p.: 162.2–168.8 °C; ^1^H NMR (400 MHz, Acetone-*d*_6_) δ 10.37 (s, 2H), 7.61 (d, *J* = 8.0 Hz, 2H), 7.57 (d, *J* = 2.8 Hz, 2H), 7.22 (d, *J* = 7.6 Hz, 2H), 7.04 (t, *J* = 8.0 Hz, 2H), 6.04 (s, 1H); ^13 ^C NMR (100 MHz, Acetone-*d*_6_) δ 134.84, 128.23, 125.75, 122.44, 121.28, 120.50, 118.61, 117.54, 112.10, 26.70; HRMS (ESI) calcd for C_18_H_11_Cl_2_N_3_ [M + Na]^+^: 362.0222, found 362.0237; IR ν (cm^−1^): 3429, 3128, 3069, 2676, 2245, 1622, 1568, 1492, 1436, 1338, 1205, 1194, 1081, 897, 785, 733, 580.

#### 4.2.7. 2,2-Bis(5-bromo-1H-indol-3-yl)acetonitrile (5 g)

Rf = 0.42 (petroleum ether/ethyl acetate = 1: 1); White solid; Yield 76%; m.p.: 219.6–223.7 °C; ^1^H NMR (400 MHz, Acetone-*d*6) δ 10.54 (s, 2H), 7.84 (d, *J* = 0.8 Hz, 2H), 7.54 (d, *J* = 2.4 Hz, 2H), 7.43 (d, *J* = 8.8 Hz, 2H), 7.27 (dd, *J* = 1.6, 8.4 Hz, 2H), 6.00 (s, 1H); ^13 ^C NMR (100 MHz, DMSO-*d*6) δ 135.27, 126.87, 125.49, 124.18, 120.61, 120.33, 114.05, 111.59, 108.89, 24.79; HRMS (ESI) calcd for C_18_H_11_Br_2_N_3_ [M + H]^+^: 429.9372, found 429.9371; IR ν (cm^−1^): 3425, 2244, 1709, 1567, 1458, 1419, 1337, 1287, 1263, 1136, 1099, 884, 794, 736, 581.

#### 4.2.8. 2,2-Bis(2-phenyl-1H-indol-3-yl)acetonitrile (5 h)

Rf = 0.69 (petroleum ether/ethyl acetate = 1: 1); Yellow solid; Yield 60%; m.p.: 112.0–117.9 °C; ^1^H NMR (400 MHz, Acetone-*d*_6_) δ10.67 (s, 2H), 7.93 (d, *J* = 8.0 Hz, 2H), 7.53–7.50 (m, 6H), 7.35–7.27 (m, 6H), 7.21 (t, *J* = 7.2 Hz, 2H), 7.07(t, *J* = 7.6 Hz, 2H), 6.06 (s, 1H); ^13 ^C NMR (100 MHz, Acetone-*d*_6_) δ 137.32, 137.14, 132.74, 129.45, 129.28, 128.96, 128.05, 122.98, 121.46, 120.72, 120.61, 112.40, 107.14, 27.07; HRMS (ESI) calcd for C_30_H_21_N_3_ [M + Na]^+^: 446.1628, found 446.1640; IR ν (cm^−1^): 3398, 3058, 2240, 2192, 1890, 1605, 1579, 1488, 1455, 1424, 1340, 1309, 1265, 1246, 1159, 1027, 743, 698, 504.

#### 4.2.9. 2,2-Bis(2-methyl-1H-indol-3-yl)acetonitrile (5i)

Rf = 0.52 (petroleum ether/ethyl acetate = 1: 1); Red solid; Yield 72%; m.p.: 231.2–236.6 °C; ^1^H NMR (400 MHz, Acetone-*d*_6_) δ 10.17 (s, 2H), 7.63 (d, *J* = 7.6 Hz, 2H), 7.33 (d, *J* = 8.0 Hz, 2H), 7.05 (td, *J* = 0.8, 7.2 Hz, 2H), 6.97 (td, *J* = 0.8, 7.6 Hz, 2H), 5.95 (s, 1H), 2.45 (s, 6H); ^13 ^C NMR (100 MHz,Acetone-*d*_6_) δ 136.29, 133.60, 128.17, 121.75, 121.23, 119.99, 118.92, 111.57, 106.04, 25.13, 12.09; HRMS (ESI) calcd for C_20_H_17_N_3_ [M + Na]^+^: 322.1315, found 322.1327; IR ν (cm^−1^): 3396, 3056, 2924, 2238, 2191, 1705, 1620, 1585, 1489, 1460, 1428, 1304, 1263, 1246, 1157, 1017, 743.

#### 4.2.10. 2,2-Bis(4-methyl-1H-indol-3-yl)acetonitrile (5j)

Rf = 0.61 (petroleum ether/ethyl acetate = 1: 1); White solid; Yield 81%; m.p.: 246.2–250.1 °C; ^1^H NMR (400 MHz, Acetone-*d*_6_) δ 10.32 (s, 2H), 7.31 (d, *J* = 8.0 Hz, 2H), 7.101–7.095 (m, 2H), 7.05 (t, *J* = 8 Hz, 2H), 6.46 (d, *J* = 6.8 Hz, 2H), 6.36 (s, 1H), 2.66 (s, 6H); ^13 ^C NMR (100 MHz,Acetone-*d*_6_) δ 138.63, 130.60, 125.37, 125.11, 123.15, 122.55, 122.03, 113.34, 110.74, 30.67, 20.14; HRMS (ESI) calcd for C_20_H_17_N_3_ [M + H]^+^: 300.1495, found 300.1497; IR ν (cm^−1^): 3406, 3056, 2926, 2237, 1958, 1752, 1617, 1577, 1459, 1408, 1337, 1263, 1156, 1116, 1048, 764, 742.

#### 4.2.11. 2,2-Bis(5-methyl-1H-indol-3-yl)acetonitrile(5k)

Rf = 0.60 (petroleum ether/ethyl acetate = 1: 1); White solid; Yield 67%; m.p.: 205.1–210.1 °C; ^1^H NMR (400 MHz, Acetone-*d*_6_) δ 10.20(s, 2H), 7.45 (s, 2H), 7.33 (d, *J* = 7.6 Hz, 4H), 6.98 (d, *J* = 8.4 Hz, 2H), 5.86 (s, 1H), 2.37 (s, 6H); ^13 ^C NMR (100 MHz,Acetone-*d*_6_) δ 136.43, 128.99, 126.96, 124.66, 124.47, 121.20, 119.28, 112.40, 110.67, 26.65, 21.71; HRMS (ESI) calcd for C_20_H_17_N_3_ [M + Na]^+^: 322.1315 found 322.1329; IR ν (cm^−1^): 3406, 3123, 2922, 2857, 2235, 1626, 1582, 1484, 1422, 1341, 1245, 1096, 1040, 800, 736, 593.

#### 4.2.12. 2,2-Bis(6-methyl-1H-indol-3-yl)acetonitrile (5 l)

Rf = 0.75 (petroleum ether/ethyl acetate = 1: 1); White solid; Yield 70%; m.p.: 178.4–183.7 °C; ^1^H NMR (400 MHz, Acetone-*d*_6_) δ 10.13 (s, 2H), 7.52 (d, *J* = 8.4 Hz, 2H), 7.31 (d, *J* = 2.4 Hz, 2H), 7.25 (s, 2H), 6.88 (d, *J* = 8.4 Hz, 2H), 5.87 (s, 1H), 2.40 (s, 6H); ^13 ^C NMR (100 MHz,Acetone-*d*_6_) δ 138.45, 132.30, 124.62, 123.83, 121.89, 121.18, 119.40, 112.46, 111.04, 26.79, 21.79; HRMS (ESI) calcd for C_20_H_17_N_3_ [M + Na]^+^: 322.1315, found 322.1328; IR ν (cm^−1^): 3408, 3130, 2921, 2859, 2241, 1711, 1628, 1548, 1454, 1341, 1264, 1246, 1096, 1041, 801, 735, 595.

#### 4.2.13. 2,2-Bis(7-methyl-1H-indol-3-yl)acetonitrile (5 m)

Rf = 0.21 (petroleum ether/dichloromethane = 2: 5); White solid; Yield 89%; m.p.: 214.1–219.4 °C; ^1^H NMR (400 MHz, Acetone-*d*_6_) δ 10.32 (s, 2H), 7.48–7.45 (m, 2H), 7.37 (d, *J* = 2.8 Hz, 2H), 6.96–6.92 (m, 4H), 5.91 (s, 1H), 2.49 (s, 6H); ^13 ^C NMR (100 MHz,Acetone-*d*_6_) δ 137.49, 126.38, 124.19, 123.39, 121.89, 121.11, 120.41, 117.43, 111.70, 26.88, 16.94; HRMS (ESI) calcd for C_20_H_17_N_3_ [M + H]^+^: 300.1495, found 300.1495; IR ν (cm^−1^): 3409, 3118, 2922, 2856, 2247, 1835, 1660, 1614, 1437, 1343, 1225, 1122, 1067, 787, 744, 585.

#### 4.2.14. 2,2-Bis(5-fluoro-2-methyl-1H-indol-3-yl)acetonitrile (5n)

Rf = 0.78 (petroleum ether/ethyl acetate = 1: 1); Yellow solid; Yield 80%; m.p.: 183.4–188.0 °C; ^1^H NMR (400 MHz, Acetone-*d*_6_) δ10.34 (s, 2H), 7.35 (q, *J* = 4.4 Hz, 2H), 7.27 (dd, *J* = 2.8, 10.4 Hz, 2H), 6.87 (dt, *J* = 2.4, 9.2 Hz, 2H), 5.94 (s, 1H), 2.47 (s, 6H); ^13 ^C NMR (100 MHz, Acetone-*d*_6_) δ 158.45 (d, *J* = 230.7 Hz), 136.12, 132.85, 128.32 (d, *J* = 10.1 Hz), 120.89, 112.61 (d, *J* = 9.6 Hz), 109.68 (d, *J* = 26 Hz), 105.87 (d, *J* = 4.5 Hz), 103.69 (d, *J* = 24.4 Hz), 25.09, 12.17; HRMS (ESI) calcd for C_20_H_15_F_2_N_3_ [M + Na]^+^: 358.1126, found 358.1140; IR ν (cm^−1^): 3410, 2953, 2925, 2855, 2361, 2238, 2191, 1708, 1630, 1583, 1486, 1455, 1265, 1178, 1129, 1089, 847, 798, 601.

#### 4.2.15. 2,2-Bis(2,5-dimethyl-1H-indol-3-yl)acetonitrile (5o)

Rf = 0.22 (petroleum ether/ethyl acetate = 4: 1); White solid; Yield 66%; m.p.: 231.2–236.4 °C; ^1^H NMR (400 MHz, Acetone-*d*_6_) δ 9.97 (s, 2H), 7.42 (s, 2H), 7.16 (d, *J* = 8.4 Hz, 2H), 6.85 (dd, *J* = 1.2, 8.4 Hz, 2H), 5.83 (s, 1H), 2.37 (s, 6H), 2.30 (s, 6H); ^13 ^C NMR (100 MHz, Acetone-*d*_6_) δ 134.63, 133.55, 128.65, 128.49, 123.26, 121.31, 118.76, 111.26, 105.62, 25.12, 21.85, 12.15; HRMS (ESI) calcd for C_22_H_21_N_3_ [M + Na]^+^: 350.1682, found 350.1643; IR ν (cm^−1^): 3395, 2920, 2236, 1958, 1716, 1587, 1310, 1247, 1028, 869, 735, 591.

#### 4.2.16. 2,2-Bis(5-methoxy-1H-indol-3-yl)acetonitrile (5p)

Rf = 0.69 (petroleum ether/ethyl acetate = (1: 1); Greyish white solid; Yield 84%; m.p.: 118.5–123.0 °C; ^1^H NMR (400 MHz, DMSO-*d*_6_) δ 10.99 (s, 2H), 7.34 (d, *J* = 2.4 Hz, 2H), 7.30 (d, *J* = 8.8 Hz, 2H), 7.05 (d, *J* = 2.4 Hz, 2H), 6.77 (dd, *J* = 2.8, 9.2 Hz, 2H), 5.98 (s, 1H), 3.70 (s, 6H); ^13 ^C NMR (100 MHz, DMSO-*d*_6_) δ 153.17, 131.61, 125.63, 124.31, 120.71, 112.54, 111.44, 109.10, 100.58, 55.35, 25.06; HRMS (ESI) calcd for C_20_H_17_N_3_O_2_ [M + Na]^+^: 354.1213, found 354.1228; IR ν (cm^−1^): 3407, 3055, 2937, 2832, 2240, 1715, 1626, 1586, 1487, 1214, 1174, 1027, 925, 802, 737, 623.

#### 4.2.17. Dimethyl 3,3'-(cyanomethylene)bis(1H-indole-5-carboxylate) (5q)

Rf = 0.21 (petroleum ether/ethyl acetate = 4: 1); Red solid; Yield 26%; m.p.: 259.6–266.9 °C; ^1^H NMR (400 MHz, DMSO-*d*6) δ 11.61 (s, 2H), 8.31 (s, 2H), 7.78 (dd, *J* = 1.2, 8.4 Hz, 2H), 7.53–7.48 (m, 4H), 6.37 (s, 1H), 3.82 (s, 6H); ^13 ^C NMR (100 MHz, DMSO-*d*6) δ 167.07, 139.27, 125.92, 124.83, 122.71, 121.20, 120.62, 120.50, 112.08, 110.70, 51.80, 24.86; HRMS (ESI) calcd for C_22_H_17_N_3_O_4_ [M + H]^+^:388.1292, found 388.1292; IR ν (cm^−1^): 3357, 2959, 2933, 2874, 1723, 1619, 1436, 1285, 1122, 1074, 747.

#### 4.2.18. 2,2-Bis(1-methyl-1H-indol-3-yl)acetonitrile (5r)

Rf = 0.25 (petroleum ether/ethyl acetate =4: 1); White solid; Yield 84%; m.p.: 70.3–74.6 °C; ^1^H NMR (400 MHz,DMSO-*d*_6_) δ 7.62 (d, *J* = 7.6 Hz, 2H), 7.42 (t, *J* = 8.4 Hz, 4H), 7.19 (t, *J* = 8.0 Hz, 2H), 7.05 (t, *J* = 8.0 Hz, 2H), 6.10 (s, 1H), 3.75 (s, 6H); ^13 ^C NMR (100 MHz,DMSO-*d*_6_) δ 136.94, 127.90, 125.49, 121.68, 120.65, 119.08, 118.70, 110.08, 108.60, 32.41, 24.93; HRMS (ESI) calcd for C_20_H_17_N_3_ [M + Na]^+^: 322.1315, found 322.1326; IR ν (cm^−1^): 3055, 2934, 2239, 1717, 1642, 1615, 1529, 1472, 1374, 1332, 1129, 1013, 742.

#### 4.2.19. 2,2-Bis(5-bromo-1-methyl-1H-indol-3-yl)acetonitrile (5 s)

Rf = 0.71 (petroleum ether/ethyl acetate = 1: 1); White solid; Yield 77%; m.p.: 177.7–181.7 °C; ^1^H NMR (400 MHz, Acetone-*d*_6_) δ 7.78 (s, 2H), 7.40 (t, *J* = 3.6 Hz, 4H), 7.31 (dd, *J* = 2.0, 8.8 Hz, 2H), 5.99 (s, 1H), 3.84 (s, 6H); ^13 ^C NMR (100 MHz,Acetone-*d*_6_) δ 136.48, 128.00, 126.09, 125.53, 121.81, 120.57, 114.49, 113.12, 110.15, 30.67, 26.35; HRMS (ESI) calcd for C_20_H_15_Br_2_N_3_ [M + Na]^+^: 479.9504, found 479.9522; IR ν (cm^−1^): 3116, 3071, 2923, 2826, 2241, 1725, 1545, 1475, 1376, 1295, 1144, 1047, 856, 793, 737, 706.

#### 4.2.20. 2,2-Bis(1-methyl-1H-pyrrol-3-yl)acetonitrile (6a)

Rf = 0.41 (petroleum ether/ethyl acetate = 4: 1); Brown oily liquid; Yield 56%; ^1^H NMR (400 MHz, Acetone-*d*6) δ 6.72 (t, *J* = 2.4 Hz, 2H), 6.01–5.99 (m, 2H), 5.74 (s, 1H), 3.57 (s, 6H); ^13 ^C NMR (100 MHz,Acetone-*d*6) δ 125.11, 124.90, 118.75, 109.76, 107.67, 34.18, 28.67; HRMS (ESI) calcd for C_12_H_13_N_3_ [M + H]^+^: 200.1182, found 200.1182; IR ν (cm^−1^): 3105, 2947, 2234, 2177, 1597, 1492, 1305, 1092, 772, 755, 720, 607.

#### 4.2.21. 2,2-Bis(5-methyl-1H-pyrrol-3-yl)acetonitrile (6 b)

Rf = 0.25 (petroleum ether/ethyl acetate = 4: 1); Brown oily liquid; Yield 58%; ^1^H NMR (400 MHz, Acetone-*d*6) δ9.77 (s, 2H), 5.94 (s, 2H), 5.69 (s, 2H), 5.39 (s, 1H), 2.17 (s, 6H); ^13 ^C NMR (100 MHz, Acetone-*d*6) δ 129.50, 124.00, 119.56, 108.10, 106.63, 12.16; HRMS (ESI) calcd for C_12_H_13_N_3_ [M + H]^+^: 200.1182, found 200.1182; IR ν (cm^−1^): 3336, 2924, 2854, 2196, 1958, 1688, 1588, 1489, 1399, 1042, 776.

#### 4.2.22. 2,2-Di(1H-pyrrol-3-yl)acetonitrile (6c)

Rf = 0.21 (petroleum ether/ethyl acetate = 4: 1); Brown oily liquid; Yield 62%; ^1^H NMR (400 MHz, Acetone-*d*6) δ 10.13 (s, 2H), 6.78 (d, *J* = 1.2 Hz, 2H), 6.11 (d, *J* = 1.2 Hz, 2H), 6.05 (d, *J* = 2.8 Hz, 2H), 5.59 (s, 1H); ^13 ^C NMR (100 MHz, CDCl_3_) δ 122.79, 119.44, 117.71, 109.04, 108.32, 29.99; HRMS (ESI) calcd for C_10_H_9_N_3_ [M + H]^+^:172.0869, found 172.0869; IR ν (cm^−1^): 3364, 2924, 2853, 2196, 1958, 1716, 1586, 1394, 1097, 1040, 730.

#### 4.2.23. Diethyl 2,2’-(((cyanomethylene)bis(4,1-phenylene))bis(methylazanediyl))diacetate (7a)

Rf = 0.21 (petroleum ether/ethyl acetate = 4: 1); Light yellow liquid; Yield 83%; ^1^H NMR (400 MHz, Acetone-*d*6) δ7.22–7.18 (m, 4H), 6.73–6.69 (m, 4H), 5.23 (s, 1H), 4.14–4.09 (m, 8H), 3.04 (s, 6H), 1.20 (t, *J* = 7.2 Hz, 6H); ^13 ^C NMR (100 MHz, Acetone-*d*6) δ 171.07, 149.60, 129.06, 126.07, 121.60, 113.15, 61.10, 54.23, 40.85, 39.55, 14.58; HRMS (ESI) calcd for C_24_H_29_N_3_O_4_ [M + H]^+^: 424.2231, found 424.2231; IR ν (cm^−1^): 2982, 1743, 1601, 1518, 1373, 1290, 1185, 1117, 1028, 946, 835, 770.

#### 4.2.24. Diethyl 2,2’-(((cyanomethylene)bis(4,1-phenylene))bis(azanediyl))diacetate (7 b)

Rf = 0.61 (petroleum ether/ethyl acetate = 1: 1); White solid; Yield 58%; m.p.: 87.5–93.4 °C; ^1^H NMR (400 MHz, CDCl_3_) δ 7.11 (d, *J* = 8.4 Hz, 4H), 6.56 (d, *J* = 8.4 Hz, 4H), 4.94 (s, 1H), 4.37 (s, 2H), 4.24 (q, *J* = 6.8 Hz, 4H), 3.87 (s, 4H), 1.29 (t, *J* = 8.0 Hz, 6H); ^13 ^C NMR (100 MHz, CDCl_3_) δ 170.82, 146.65, 128.62, 125.71, 120.50, 113.16, 61.36, 45.62, 40.94, 14.13; HRMS (ESI) calcd for C_22_H_25_N_3_O_4_ [M + H]^+^: 396.1918, found 396.1918; IR ν (cm^−1^): 3398, 2983, 2936, 2241, 1739, 1614, 1522, 1374, 1211, 1024, 823.

#### 4.2.25. Dimethyl 2,2’-(((cyanomethylene)bis(4,1-phenylene))bis(azanediyl))diacetate (7c)

Rf = 0.39 (petroleum ether/ethyl acetate = 4: 1); White solid; Yield 61%; m.p.:135.7–140.8 °C;^1^H NMR (400 MHz, CDCl_3_) δ 7.12 (d, *J* = 8.4 Hz, 4H), 6.56 (d, *J* = 8.4 Hz, 4H), 4.94 (s, 1H), 4.16 (s, 2H), 3.89 (s, 4H), 3.77 (s, 6H); ^13 ^C NMR (100 MHz, CDCl_3_) δ 171.35, 146.59, 128.64, 125.83, 120.45, 113.21, 52.23, 45.46, 40.94; HRMS (ESI) calcd for C_20_H_21_N_3_O_4_ [M + H]^+^: 368.1605, found 368.1605; IR ν (cm^−1^): 3390, 2900, 2239, 1727, 1614, 1520, 1436, 1361, 1215, 1179, 991, 810.

### 4.3. α-Glucosidase inhibition activities and mechanism assay

The α-glucosidase inhibitory activity of compounds **5a ∼ c**, **6a ∼ c**, and **7a ∼ c** were performed according to our previous report.^30^^,^^31^ The 10 μL of α-glucosidase enzyme (final concentration 0.1 U/mL) and 10 μL of test compounds (dissolved in DMSO) were added into 130 μL of phosphate buffer (0.1 M, pH 6.8), followed 10 min incubation at 37 °C. Then 50 μL of PNPG (final concentration 0.25 mM) was added, and mixture was continuely incubated for 20 min at 37 °C. The absorbance at 405 nm was measured using Multimodel Reader. The percentage of enzyme inhibition was calculated: % Inhibition = [(A_1_– A_0_)/A_0_] × 100%, where A_1_ was the absorbance with the test compound, and A_0_ was the absorbance without the test compound. The IC_50_ value of compound was obtained from the plot of inhibition percentage *vs* test compound at different concentrations. Acarbose was used as the positive control. The experiment was performed in duplicate.

The inhibition mechanism of compounds **5 g**, **5e**, and **5 h** was analysed using similar above method. The enzyme inhibitory kinetics was detected using plots of enzyme concentration *vs* remaining enzyme activity at different inhibitor concentrations, and the substrate inhibitory kinetics was obtained from Lineweaver-Burk plot of remaining enzyme activity *vs* substrate concentration in the presence of different inhibitor concentrations.^32^

### 4.4. α-Amylase *inhibition activities and mechanism assay*

The α-amylase inhibitory activity of all tested compounds was performed according to previous report.^33^^,^^34^ A 10 μL of α-amylase enzyme solution (final concentration 0.25 U/mL), 10 μL of test compounds (dissolved in DMSO), and 80 μL of phosphate buffer (20 mM, pH 6.9) were mixed and incubated for 10 min at 37 °C. Next, 100 μL starch solution (final concentration 0.5%) was added into the mixture followed by an incubation of 10 min. After 100 μL DNS (containing 1 M Potassium sodium tartrate and 48 mM 3,5-Dinitrosalicylic acid) was added, the mixture was kept incubated in boiling water for 15 min. Finally, the absorbance was measured at 540 nm after dilution of solution by adding 900 μL distilled water. The percentage of enzyme inhibition was calculated: % Inhibition = [(A_1_– A_0_)/A_0_] × 100%, where A_1_ was the absorbance with the test compound, and A_0_ was the absorbance without the test compound. The IC_50_ value of compound was obtained from the plot of inhibition percentage *vs* test compound at different concentrations. Acarbose was used as the positive control. The experiment was performed in duplicate.

The inhibition mechanism of compounds **5 g**, **5 h**, and **5S** was also analysed. The enzyme inhibitory kinetics was detected using plots of enzyme concentration *vs* remaining enzyme activity at different inhibitor concentrations, and the substrate inhibitory kinetics was obtained from Lineweaver-Burk plot of remaining enzyme activity *vs* substrate concentration in the presence of different inhibitor concentrations.

### 4.5. Molecular docking

Molecular docking was conducted to explore the interaction of inhibitors with α-glucosidase and *α-*amylase using Sybyl (Version 2.1.1, Tripos, US) according to our previous report.^33^ The crystal structure of α-glucosidase (PDB: 3AJ7)^33^^,^^35^ and α-amylase (PDB: 3BAJ)^36^^,^^37^ were obtained from the Protein Data Bank. Compounds were prepared with addition of hydrogen atoms, addition of charge with Gasteiger-Hückle mode, and energy minimisation. Then the protein was prepared by procedure of removing H_2_O, fixing side chain amides, and adding hydrogens. The active site of α-glucosidase was simulated out using automatic mode. The active site of α-amylase was simulated out using Ligen mode. Then, the docking simulations between compounds and α-glucosidase or α-amylase were carried out with the default format of Pymol program.

### 4.6. Cell cytotoxicity assay

The 3T3-L1 cells or HepG2 cells were cultured in DMEM medium containing 10% foetal bovine serum, 100 U/mL penicillin and 0.1 mg/mL streptomycin in a humidified incubator with a 5% CO_2_ atmosphere at 37 °C. Cells in the logarithmic growth phase were used for this assay. After 5 × 10^3^ 3T3-L1 cells were seeded in 96 well plates for 24 h, compound with different concentration was added into each well for 24 h. MTT reagent (100 μL, 0.5 mg/mL) was added to each well for 4 h incubation. After the supernatant was discarded, 100 μL of DMSO was added. The absorbance was measured at 490 nm. Each sample was performed in 3 parallel experiments.
